# Isolation of *Rickettsia typhi* from Human, Mexico

**DOI:** 10.3201/eid2008.130095

**Published:** 2014-08

**Authors:** Jorge E. Zavala-Castro, Karla R. Dzul-Rosado, Gaspar Peniche-Lara, Raúl Tello-Martín, Jorge E. Zavala-Velázquez

**Affiliations:** Universidad Autónoma de Yucatán, Mérida, México

**Keywords:** *Rickettsia typhi*, human case, Rickettsia isolation, centrifugation cell culture plate technique, bacteria, Mexico

**To the Editor:** Murine typhus is a febrile illness caused by *Rickettsia typhi*. The clinical manifestations are nonspecific, and the signs and symptoms resemble those of several other febrile illnesses. Murine typhus can be a self-limiting infection; however, it should be diagnosed and treated because complications and even death can result (*1*). In Mexico, particularly in Yucatan State, cases of murine typhus in humans and high prevalence of antibodies in healthy blood donors have been reported (*2*,*3*). In 2012, we isolated *R. typhi* from a human patient in southeastern Mexico by using a simple and effective method, an adaptation of the centrifugation shell vial method to cell culture plates.

The patient, a 23-year-old man from Dzibzantun (21°15′00″N, 89°03′00″W), in the northeastern part of Yucatan State, was referred for possible diagnosis of rickettsial infection. He had a low-grade fever (37.6°C) and a maculopapular rash on the thorax and upper and lower extremities. The patient reported having cats in the house, but no fleas or ticks were observed. Clinical laboratory findings were within reference ranges. Test results were negative for dengue virus, but the Weil-Felix (Proteus OX19) test result was positive (titer 1:164). Single-step PCR amplification was performed by using genus-specific primers for the 17-kDa lipoprotein and the citrate synthase gene (*gltA*), as described previously, to obtain amplicons of 434 bp and 380–385 bp (*4*). PCR was positive for *R. typhi*, and 100 mg of oral doxycycline 2 times per day for 7 days was prescribed; the rash cleared.

We subjected 5 mL of blood to centrifugation for 1 hour at 1,000 rpm and then stored the plasma at −80°C. Blood samples from other patients were used as controls. A total of 50,000 Vero cells were grown in 8 central wells of a 24-well cell culture cluster (Corning Incorporated, Corning, NY, USA) with minimal essential medium (MEM; Biowest, Nuaillé, France) supplemented with 10% fetal bovine serum (Biowest) and incubated at 37°C with 5% CO_2_ for 48 hours to obtain 95% confluence. We then thawed 700 μL of the plasma in a 37°C water bath. The MEM was discarded, and the wells were refilled with 250 μL each of a mixture of the plasma and fresh medium at a 1:3 ratio. The plaque was covered with parafilm and centrifuged at 700 *g* for 60 minutes at 22°C. The supernatant was discarded and replaced with 1 mL of MEM supplemented with 5% fetal bovine serum, 100 U penicillin, 100 μg streptomycin, and 250 ng amphotericin B (Sigma Aldrich, St. Louis, MO, USA) and incubated at 33°C with 5% CO_2_.

On day 3 after sample inoculation, the antimicrobial drug–containing medium was removed and replaced with MEM without antimicrobial drug and supplemented with 5% fetal calf serum (HyClone Laboratories, Inc., South Logan, UT, USA). Medium was changed every 3 days until day 15. A cell sample from each well was tested for infection at days 9 and 15 by using Gimenez stain and PCR with *17 kDa* and *gltA* primers.

Gimenez staining on day 15 yielded numerous red-stained bacteria in the cytoplasm of Vero cells in the 8 wells used. A single scraping of the cells from the positive wells was inoculated onto confluent layers of Vero cells, which enabled establishment of the isolate.

Three PCR amplicons of the *17kDa*– and *gltA–*specific primers (*4*–*6*) from positive wells were fully sequenced. After removing primer sequences, we compared amplicon sequences by conducting a gapped BLAST 2.0 (http://blast.st-va.ncbi.nlm.nih.gov/Blast.cgi) search of the GenBank database; the *17-kDa* (accession no. JX198507) and *gltA *(accession no. KC469611) gene fragment sequences showed 100% identity with *R. typhi* strain Wilmington (accession no. AE017197.1).

Murine typhus has been reemerging in southeastern Mexico for the past 6 years (*3*,*7*). Active epidemiologic surveillance led to early detection of human cases and opportune treatment, thereby decreasing the rate of severe illness. However, the prevalent social and cultural conditions in small villages, with close contact with domestic, peridomestic, and wild animals, facilitate the transmission of this fleaborne rickettsiosis; human infections, such as the case presented here, still occur.

We replaced shell vials with cell culture plates and isolated rickettsiae from a biological sample from a patient with acute murine typhus. The method is as simple as the shell vial centrifugation technique and is highly sensitive and easy to perform, making it an excellent choice for rickettsiae isolation when shell vials are not available.

In the United States, isolation of *R. typhi *from a human was last reported >50 years ago (*8*). The case reported here reinforces the need to extend surveillance to small towns and villages in Yucatan State. It also shows that a shell vial alternative method for *R. typhi* isolation is simple and effective.
